# Recommendations of Professor Gorgas' Works

**Published:** 1886-02

**Authors:** 


### Bibliographical.

---

Recommendations of Professor Gorgas' Works.—*From the Independent Practitioner, Dr. W. Barrett, Editor.*—" In the last volume of this journal we received the first edition of this work, and now we are called upon to notice the second. It is a very unusual event in the history of any medical book, when the second edition is so soon called for. When the first appeared we predicted that it would meet with favor, and the unusual sales have verified the not very hazardous forecast. The second edition is a considerable improvement upon the first. Sixty-eight pages have been added, and the whole text revised. Short as has been the time since the first edition, there were already a number of new remedies offered to the profession, all of which, we believe have received due notice. Gorgas' Dental Medicine has already taken its place near the head of our professional literature. It has, we believe, no equal in the English language, if in any other, as a text-book on dental medicine, and we shall look to see it adopted in all countries, where there is dental teaching, as the standard work on dental Materia Medica and Therapeutics."

---

*From the Dental Cosmos, Dr. Jas. W. White, Editor.*

" The sale within less than two years of the first edition of this volume is an indication of the need which existed for such a manual. The work has been entirely revised, and many valuable additions made to it, including a chapter on inflammation with special reference to the mucous membranes of the mouth. All the new agents used in dental practice have received notice, and the book gives evidence of conscientious, painstaking labor on the part of the author. An index to diseases and dental formulary has been added, facilitating easy reference to pathological condition and remedial indications. Additions have also been made to the dental formulæ with fuller explanations of the methods of prominent practitioners for the special employment of medicinal agents and the results of recent investigations into the properties of anæsthetic agents. The volume as a whole commends itself as an educator and as a work of reference which should be in the hands of every student and every practitioner of dentistry."

---

*From the Southern Dental Journal, Dr. B. H. Catching, Editor.*

"Second Edition, Revised and Improved of Gorgas Dental Medicine. Only a few months ago we had the pleasure of calling attention to the first edition of this valuable work. Its merits we highly appreciated and spoke of. Even under the most favorable criticisms and circumstances, we did not think that so soon would we be called upon again to present its great merits in an enlarged and revised second edition. While the large sale of this work is pleasing to both author and publisher, it is no less so to this journal, as an indication of the demands of the dentists in these important branches of dental science. Truly a healthy sign. The second edition contains sixty-eight additional pages, etc., etc."

---

*From "The Dental Eclectic," S. S. Willard, D. D. S. Editor.*

"The first edition of this most excellent and indispensable work was issued not quite two years ago, and the fact that it has already reached its second edition is in itself a sufficient guarantee of its popularity and value to the profession. Up to the date of issue of Dr. Gorgas' work the dental profession had suffered from a scarcity of works of this character, and its members were compelled to look to works on general Materia Medica and Therapeutics for their needs· Dr. J. W. White published a small work on the subject in 1868, and about this time or later. one was issued in London by Dr. J. Stocken and Thomas Geddes. There is a marked improvement in the second edition. The whole work has been entirely revised and sixty-eight pages added to the volume. Among the added chapters are admirable ones on "Inflammation with special reference to the Mucous Membrane of the Mouth," etc., etc., etc. An interesting and valuable portion of the work is that containing a diagnosis and synopsis of treatment of mouth affections, etc., etc., etc. The volume shows careful work throughout, and a thorough knowledge of the subjects treated. It has already gained a place in the fronk rank of dental literature, and no dental office or library is complete without this standard work on Dental Materia Medica and Therapeutics."

---

*From The Dental Advertiser, Theodore G. Lewis, D. D. S., Editor.*

"The necessity for a second edition of Gorgas' Dental Medicine is gratifying evidence of the estimation in which the work is held by the profession. We have found the first edition of inestimable value, and have had occasion to refer to it more frequently than to any other work in our possession, and always with gratifying results. The present edition has been thoroughly revised and from the author's preface we learn that among the additions are, etc., etc., etc., making the most complete work on dental medicine ever published."

---

*From The Dental Practitioner, Chas. E. Pike, D. D. S., and L. Ashley Faught, D. D. S., Editors.*

"Gorgas' Dental Medicine—The author of this work having already given to the profession of dentistry "Harris' Principles and Practice of Dentistry," and "Harris' Dictionary of Medical Terminology and Dental Surgery," now by its production closes up an important gap in all dental libraries. The want has long been felt, by not a few, of a work which shall deal with the medicines used in dentistry from a purely dental standpoint; and yet be sufficiently comprehensive in its construction to embrace enough general, practical information to enable a practitioner to use his drugs with confidence and power. We believe that the work of Dr. Gorgas, as it comes to us in this second edition, very largely accomplishes this object, and feel that no dentist or dental student can afford to be without one, etc., etc., etc."

